# Multiplex networks-based directed graph neural network for cancer driver gene identification

**DOI:** 10.1371/journal.pcbi.1014275

**Published:** 2026-05-14

**Authors:** Pingting Li, Minzhu Xie

**Affiliations:** College of Information Science and Engineering, Hunan Normal University, Changsha, China; Ocean University of China, CHINA

## Abstract

Identifying cancer driver genes is crucial in precision oncology. Most existing methods rely on a single interaction network to capture gene relationships. However, with the increasing availability of multi-omics and biological network data, integrating multiplex networks offers a more comprehensive representation of the complex and directional regulatory interactions among genes. Moreover, the number of validated cancer driver genes remains small compared with the vast number of unlabeled genes, leading to label scarcity and class imbalance. To address these limitations, we propose a multiplex networks-based directed graph neural network (MNDGNN). The model learns gene representations on multiplex networks with multi-omics data through directed graph convolution, which integrates neighbor diversity and degree diversity. We also incorporate data augmentation combining positive-sample augmentation with negative-sample inference to mitigate label scarcity. Experimental results show that the proposed method achieves better predictive performance and robustness than existing state-of-the-art methods. The predicted cancer driver genes are significantly enriched in cancer-related pathways and exhibit extensive interactions with known cancer driver genes, offering a new perspective for cancer driver gene discovery and the design of therapeutic strategies.

## Author summary

Cancer genomes often contain many mutations, but only a small fraction actively promote tumor growth. Therefore, distinguishing driver mutations from the vast background of passenger mutations is a critical task for understanding disease mechanisms and developing targeted therapies. Although large-scale sequencing has enabled the discovery of hundreds of cancer driver genes, many of these genes remain difficult to interpret because relevant evidence is scattered across different data types and biological interaction networks, and only a limited set has been experimentally validated. In this study, we develop a computational approach that integrates multi-omics data with multiplex biological interaction networks, rather than relying on a single network. We also incorporate directionality in regulatory relationships to better reflect how signals propagate through gene networks. In addition, we employ a data augmentation strategy to facilitate effective learning under label scarcity. Our method improves predictive performance over existing approaches and prioritizes candidate cancer driver genes that are strongly connected to known cancer driver genes and enriched in cancer-relevant pathways, providing a practical shortlist for downstream experimental validation and therapeutic target discovery.

## Introduction

Cancer is a class of diseases characterized by the uncontrolled proliferation of somatic cells. It arises from driver mutations in the human genome, which confer a selective growth advantage to the affected cells and thereby promote tumorigenesis. Genes harboring such mutations are called cancer driver genes (CDGs) [1–4]. As cancer driver genes are under positive selection and contribute to the disruption of key cellular functions, identifying these genes facilitates cancer diagnosis and targeted therapies, and plays a critical role in precision oncology [1].

In recent years, computational methods for identifying cancer driver genes have increased. This growth relies on rich multi-omics resources from databases such as TCGA and biological networks modeling complex molecular interactions. Meanwhile, deep learning has advanced rapidly. Graph neural networks (GNNs) show a strong ability to capture topological information from a network, leading to their widespread application in this field. EMOGI [5] is an explainable method based on graph convolutional networks (GCNs) for cancer driver gene identification through the integration of multi-omics data and protein-protein interaction (PPI) network. MTGCN [6] introduces a multi-task learning graph convolutional network based on a ChebNet [7] variant, enhancing cancer driver gene identification through joint optimization of node classification and link prediction tasks. ECD-CDGI [8] primarily employs an encoder based on energy-constrained diffusion and attention mechanisms, effectively capturing complex gene dependencies. In deepCDG [9], a shared-parameter GCN encoder with an attention layer supports cross-omic integration to improve cancer driver gene identification. DGMP [10] predicts cancer driver genes by integrating a directed graph convolutional network and a multilayer perceptron to learn gene features from a gene regulatory network and multi-omics data.

However, most current methods are limited to analyzing individual networks, capturing only a single type of interaction. This not only overlooks the multifaceted functions of genes across different biological regulatory processes, but may also lead to an overrepresentation of network-specific noise. Recently, several models integrating multiplex networks have been proposed to identify cancer driver genes. MRNGCN [11] leverages three gene relationship networks to propose an identification method integrating a heterogeneous graph convolutional network with a self-attention mechanism. MMGN [12] integrates multiplex networks and multi-omics data through graph neural networks with negative sample inference to identify cancer driver genes. Both MRNGCN and MMGN represent gene regulatory processes and signaling pathways as undirected networks. However, since these processes are inherently directional, such representations may result in the loss of regulatory logic and reduced prediction accuracy [13].

While some studies, such as DGMP, have employed a directed graph convolutional network to model gene regulatory networks, these approaches generally apply uniform, layer-wise directional weights. This results in equal importance being assigned to both incoming and outgoing messages, disregarding variations in local structure. Furthermore, degree information is often reduced to a simple normalization factor. Consequently, these methods fail to account for node-level differences in the importance of directional neighbors and overlook the structural insights provided by in-degree and out-degree. This ultimately limits the model’s ability to capture fine-grained directional weighting dynamics [14].

Regarding the training data, although resources like the Network of Cancer Genes (NCG) [15] provide known cancer driver genes, their number is relatively small compared to the quantity of unlabeled genes. Furthermore, reliable datasets for non-cancer driver genes (NCDGs) are currently unavailable. This label imbalance presents challenges for model training and validation, potentially affecting classification performance [13].

To address these challenges, we propose the MNDGNN model for cancer driver gene identification. Our approach leverages multiplex networks to capture diverse types of molecular interactions and reduce noise associated with any single network. Furthermore, it employs a directed graph neural network that incorporates both neighbor diversity and degree diversity to enable fine-grained modeling of directional information. This enhances node discriminability and allows for more comprehensive use of information from multiplex biological networks. To mitigate label imbalance caused by the limited availability of known cancer driver genes and the absence of confirmed non-cancer driver genes, we introduce a data augmentation strategy that combines positive-sample augmentation with negative-sample inference. Overall, the main contributions of this paper are summarized as follows:

(1) We design a multiplex networks-based directed graph convolutional network (MDGCN). Each convolutional layer of MDGCN captures neighbor diversity and degree diversity to extract directional information of nodes in biological networks, effectively integrating both directed and undirected multiplex networks.
(2) During data augmentation, we employ low information entropy (IE) and radial basis function (RBF) [16] spectral clustering for positive sample enhancement. Additionally, an internal contrastive learning (ICL) [17] model is utilized for negative sample inference to maintain class balance.
(3) The experimental results demonstrate that MNDGNN outperforms other state-of-the-art methods in terms of AUROC, AUPRC, and F1 score on the pan-cancer dataset. It not only identifies known cancer driver genes but also uncovers potential cancer driver genes and candidate therapeutic targets.

## Materials and methods

### Materials

#### Multi-omics data.

We collected pan-cancer multi-omics data for 16 cancer types from The Cancer Genome Atlas [18] (https://portal.gdc.cancer.gov/), encompassing gene mutation, DNA methylation, and gene expression data. For each cancer type, the mutation frequency of a gene was calculated as the number of non-silent single nucleotide variants in that gene divided by its exonic gene length. The extent of DNA methylation alteration was defined as the average difference in methylation signals between tumor samples and matched normal samples for a specific cancer type. The differential expression level of each gene in a given cancer type was first quantified as the $\log_{2}$ fold change between its expression values in cancer versus matched normal samples, and then averaged across all available samples. Ultimately, we concatenated these three types of omics data for each gene across all cancer types and performed z-score normalization. The known cancer driver genes, serving as positive samples, are derived from the following resources: NCG, COSMIC Cancer Gene Census [19], and DigSee [20], comprising a total of 668 genes.

#### Biological networks.

We collected six biological networks. The specific details are as follows:

(1) PPI network: The protein-protein interaction network is an undirected network built from physical or functional interactions between proteins. Sourced from the ConsensusPathDB [21], it comprises 9,691 nodes and 365,138 edges.
(2) Protein complexes network: The network is an undirected graph representation of polypeptide chains connected by disulfide bonds and other protein interactions. Based on the CORUM [22] relationships, the graph is defined by 1,810 nodes and 25,714 edges.
(3) KEGG pathway network: KEGG pathway network describes the functional relationships established through biochemical reactions and signal transduction among gene products within biological pathways. Using the Pathview [23] tool, we integrated pathways from the KEGG [24] database. This process resulted in a directed network containing 3,007 nodes and 58,946 edges.
(4) RegNetwork: Regulatory interactions of transcription factor-transcription factor (TF-TF) and transcription factor-gene (TF-gene) pairs were systematically retrieved from RegNetwork [25] database to construct a directed regulatory network comprising 9,539 TFs and genes and 75,112 regulatory edges.
(5) DawnNet: DawnNet is a directed gene association network derived from DawnRank [26]. It was created by consolidating redundant genes into single entries and combining their corresponding edges. The resulting network contains 6,153 genes and 109,580 directed regulatory relationships.
(6) Kinase-substrate network: The kinase-substrate network primarily describes the intricate relationship where kinases regulate cellular signaling and function through the phosphorylation of their substrates. We obtained the kinase-substrate dataset curated by Wiredja et al. [27] from the PhosphoSitePlus [28] database. This directed network comprises 1,934 nodes and 4,532 edges.

### Overview of MNDGNN

**Fig 1:**
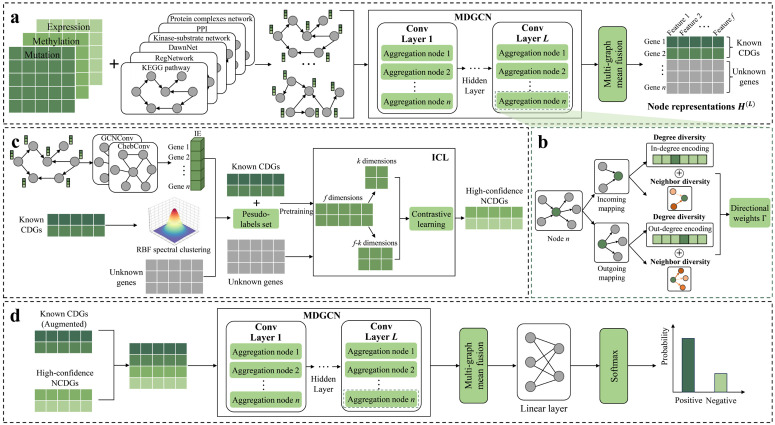
The architecture overview of MNDGNN. **(a) Data preprocessing and feature extraction:** The six biological networks incorporated with the multi-omics data are preprocessed and subsequently input into the first MDGCN to learn gene representations. **(b) Directional encoding process:** Each convolutional layer encodes directional information for node updates in the MDGCN. **(c) Data augmentation:** The model uses low information entropy and RBF-based spectral clustering to construct a pseudo-labeled set, and then applies an ICL model to identify high-confidence non-cancer driver genes. **(d) Prediction of cancer driver genes:** MNDGNN is trained on the augmented gene representations, and a linear classifier is finally applied to predict cancer driver genes.

As shown in Fig 1, MNDGNN is a deep multi-omics integration framework built on multi-network directed graph convolution for identifying cancer driver genes. The process begins with preprocessing multi-omics data and six types of biological networks, which are subsequently input into a MDGCN. The MDGCN integrates neighbor diversity and degree diversity at each convolutional layer to learn node representations with explicit directional information. At this stage, the node representations are learned only for known CDGs and unknown genes. Subsequently, a two-layer graph convolutional module is employed to compute the IE of each node, followed by RBF spectral clustering on the known CDGs to select a high-confidence pseudo-labeled positive sample set from the unknown genes. In the next step, ICL is used to extract high-confidence non-cancer driver gene representations. Finally, the augmented gene representations are delivered to another MDGCN and a linear classifier for the prediction of cancer driver genes.

### Data preprocessing

We first integrate multi-omics data, including gene mutation, DNA methylation, and gene expression, into six original biological networks by assigning these features to the corresponding gene nodes of the networks. This process yields, for each network, a multi-omics feature matrix *X* of dimensions *n* × *f*, where *n* is the number of genes and the feature dimension is *f* = 48.

To enable multiplex graphs contrastive pretraining, a corrupted multi-omics feature matrix $\tilde{X}$ is generated for each network by randomly permuting the rows of the original matrix *X*. These two sets, i.e., the original networks and their corrupted counterparts, are subsequently used as paired inputs to the MDGCN for contrastive representation learning.

### MDGCN

A MDGCN consists of several multiplex networks-based directed graph convolutional layers. Each layer takes *M* graphs (i.e., networks) as input, and undirected edges are treated as bidirectional. For the *m*-th directed graph $G^{\left(m\right)}=(V,E^{\left(m\right)},X)$, $V=\{v_{i}|i=1,2,\ldots ,n\}$ is the shared node (i.e., gene) set, $E^{\left(m\right)}\subseteq V\times V$ is the edge set of the graph, and $X\in {\mathbb{R}}^{n\times f}$ denotes the multi-omics feature matrix mentioned above. The adjacency matrix of the graph is denoted by $A^{\left(m\right)}\in \{0,1{\}}^{n\times n}$. The outgoing neighbor set of the *i*-th node *v*_*i*_ is defined as $N_{i,\to }^{\left(m\right)}=\{v_{j}|(v_{i}\to v_{j})\in E^{\left(m\right)}\}$. For graph *G*^(*m*)^, we compute a diagonal out-degree matrix and a diagonal in-degree matrix, where $\left(D_{\to }^{\left(m\right)}{)}_{i}=\sum_{j}(A_{ij}^{\left(m\right)}\right)$ and $\left(D_{\leftarrow }^{\left(m\right)}{)}_{j}=\sum_{i}(A_{ij}^{\left(m\right)}\right)$. Then a symmetric normalized directed out-neighbor matrix is defined as $S_{\to }^{\left(m\right)}=(D_{\to }^{\left(m\right)}{)}^{-\frac{1}{2}}A^{\left(m\right)}(D_{\leftarrow }^{\left(m\right)}{)}^{-\frac{1}{2}}$. Similarly, we can define $N_{i,\leftarrow }^{\left(m\right)}$and $S_{\leftarrow }^{\left(m\right)}$.

In each convolutional layer of MDGCN, the core operation is to aggregate neighborhood information for each node and update its representation based on the aggregated information. This process corresponds to the directional encoding process shown in Fig 1. Specifically, this module characterizes the directional properties of nodes through neighbor diversity and degree diversity.

#### Neighbor diversity.

Under the local homophily assumption, each node is expected to share the class label most common among either its out-neighbors or in-neighbors. Dirichlet energy [29] is a common measure of feature discrepancy between adjacent nodes. To quantify the discrepancy between individual nodes and their respective neighborhoods, we compute directional Dirichlet energy for each graph *m* at the node level. Let $H^{\left(l\right)}\in {\mathbb{R}}^{n\times f}$ denotes the node representations at layer *l*. The in-Dirichlet energy of individual nodes at the *l*-th layer is defined as follows:


$$\begin{array}{c} e_{\leftarrow }^{\left(l,m\right)}=(I+S_{\leftarrow }^{\left(m\right)})(H^{\left(l\right)}⊙H^{\left(l\right)})-2(((I+S_{\leftarrow }^{\left(m\right)})H^{\left(l\right)})⊙H^{\left(l\right)}-H^{\left(l\right)}⊙H^{\left(l\right)}), \end{array}$$ (1)

where $e_{\leftarrow }^{\left(l,m\right)}\in {\mathbb{R}}^{n\times f}$, whose *i*-th row corresponds to the in-Dirichlet energy vector of node *i*, *I* is the identity matrix, and ⊙ denotes the element-wise product. Analogously, the out-Dirichlet energy at the *l*-th layer is defined as follows:


$$\begin{array}{c} e_{\to }^{\left(l,m\right)}=(I+S_{\to }^{\left(m\right)})(H^{\left(l\right)}⊙H^{\left(l\right)})-2(((I+S_{\to }^{\left(m\right)})H^{\left(l\right)})⊙H^{\left(l\right)}-H^{\left(l\right)}⊙H^{\left(l\right)}). \end{array}$$ (2)

A larger energy value indicates a greater discrepancy between a node and its neighbors in the corresponding direction, signifying a higher likelihood of their belonging to different classes.

### Degree diversity.

Degree information can enhance a GNN’s ability to distinguish nodes. For each graph *m*, we first compute the in-degree and out-degree for each node from the adjacency matrix. Then, we use these degree values as indices to retrieve corresponding embeddings from trainable embedding matrices. Specifically, we construct two learnable embeddings for in-degree and out-degree embeddings, denoted ${\mathrm{Deg}}_{\leftarrow }^{\left(m\right)}\in {\mathbb{R}}^{n\times f}$ and ${\mathrm{Deg}}_{\to }^{\left(m\right)}\in {\mathbb{R}}^{n\times f}$.

To better characterize node representations and enhance the discrimination among structurally distinct nodes, we fuse neighbor diversity with degree diversity to obtain an adaptive directional weight for each node:


$$\begin{array}{c} \left\{ \begin{array}{cc} q_{\leftarrow }^{\left(l,m\right)} & =(-e_{\leftarrow }^{\left(l,m\right)}+{\mathrm{Deg}}_{\leftarrow }^{\left(m\right)})w_{\leftarrow }^{\left(l\right)}+b_{\leftarrow }^{\left(l\right)},\\ q_{\to }^{\left(l,m\right)} & =(-e_{\to }^{\left(l,m\right)}+{\mathrm{Deg}}_{\to }^{\left(m\right)})w_{\to }^{\left(l\right)}+b_{\to }^{\left(l\right)}, \end{array}\right. \end{array}$$ (3)

where $w_{\leftarrow }^{\left(l\right)}\in {\mathbb{R}}^{f\times 1}$ and $b_{\leftarrow }^{\left(l\right)}\in \mathbb{R}$ are learnable parameters for the incoming direction at the *l*-th layer, and $q_{\leftarrow }^{\left(l,m\right)}\in {\mathbb{R}}^{n\times 1}$. The formulation for the outgoing direction is defined analogously.

Let $\tau^{\left(l\right)}$ be a learnable adaptive temperature. The directional weights are normalized by a softmax function and the formula is as follows:


$$\begin{array}{c} \mathrm{diag}(\Gamma_{\to }^{\left(l,m\right)})=\frac{\exp(q_{\to }^{\left(l,m\right)}/\tau^{\left(l\right)})}{\exp(q_{\to }^{\left(l,m\right)}/\tau^{\left(l\right)})+\exp(q_{\leftarrow }^{\left(l,m\right)}/\tau^{\left(l\right)})}, \end{array}$$ (4)

where $\mathrm{diag}(\Gamma_{\to }^{\left(l,m\right)})\in {\mathbb{R}}^{n\times 1}$ denotes the vector of diagonal entries of the diagonal matrix $\Gamma_{\to }^{\left(l,m\right)}\in {\mathbb{R}}^{n\times n}$. This normalization yields complementary directional weights, i.e., $\Gamma_{\leftarrow }^{\left(l,m\right)}=I-\Gamma_{\to }^{\left(l,m\right)}$.

Since multiplex graphs are used as input, the multi-graph mean fusion module in Fig 1 aggregates messages from all *M* graphs and updates the node representation *H*^(*l*+1)^ at the *(l + 1)*-th layer by averaging their contributions according to Eq (5).


$$\begin{array}{c} H^{\left(l+1\right)}=\alpha H^{\left(l\right)}+\frac{1}{M}\sum_{m=1}^{M}(\Gamma_{\to }^{\left(l,m\right)}S_{\to }^{\left(m\right)}H^{\left(l\right)}W_{\to }^{\left(l\right)}+\Gamma_{\leftarrow }^{\left(l,m\right)}S_{\leftarrow }^{\left(m\right)}H^{\left(l\right)}W_{\leftarrow }^{\left(l\right)}), \end{array}$$ (5)

where the hyperparameter α retains information from the previous layer. $\Gamma_{\to }^{\left(l,m\right)}$ is diagonal with $\mathrm{diag}(\Gamma_{(1,1)\to }^{\left(l,m\right)},\;\Gamma_{(2,2)\to }^{\left(l,m\right)},\;\ldots ,\;\Gamma_{(n,n)\to }^{\left(l,m\right)})$, where each diagonal element encodes the fused neighbor and degree diversity. $W_{\to }^{\left(l\right)}$ is a learnable linear transformation matrix at the *l*-th layer used to transform the aggregation result in the outgoing direction.

In summary, for each node in each network, neighbor diversity is assessed via directional Dirichlet energy, while degree embeddings are constructed from the node’s in-degree and out-degree. These two components are fused to compute directional weights for each node. For both the original and corrupted network sets, the neighbor feature embeddings based on outgoing and incoming directions are weighted and aggregated. Finally, the resulting representations are averaged across all graphs to obtain the updated node representations.

### Data augmentation

To mitigate label scarcity and overfitting, we apply data augmentation in two stages: positive-sample augmentation and negative-sample inference.

#### Positive-sample augmentation.

We perform positive pseudo-label augmentation by combining entropy-based confidence filtering with a spectral clustering constraint based on the RBF kernel. Specifically, we first obtain node-wise class probabilities using a two-layer graph convolution, and then calculate the prediction entropy for each unlabeled node and retain low-entropy nodes as high-confidence candidates. Next, we apply a spectral clustering constraint based on the RBF kernel in the feature space, thereby filtering out outliers and promoting alignment with the labeled-positive distribution, resulting in a high-confidence set of positive pseudo-labels.

Concretely, we employ a Chebyshev convolutional layer to capture higher-order neighborhoods, followed by a GCN layer for normalized aggregation, and compute the class probabilities as follows:


$$\begin{array}{c} P=\mathrm{softmax}\;\left(D^{-\frac{1}{2}}AD^{-\frac{1}{2}}\left[\sum_{k=0}^{K}T_{k}(\hat{L})\;X\Theta_{k}\right]W\right), \end{array}$$ (6)

where *K* denotes the Chebyshev order, and $T_{k}(\cdot )$ is the Chebyshev polynomial satisfying the recurrence $T_{0}=I,\;T_{1}=\hat{L},$ and $T_{k}=2\hat{L}T_{k-1}-T_{k-2}$ for *k* ≥ 2. $\hat{L}$ is the scaled graph Laplacian. $\{\Theta_{k}\overset{K}{\underset{k=0}{\}}}$ and *W* are the learnable weight matrices of the two convolutional layers. We then compute the information entropy for each unlabeled node as follows:


$$\begin{array}{c} {ℋ}_{i}=-\sum_{c=1}^{C}p_{ic}\log p_{ic}, \end{array}$$ (7)

where *p*_*ic*_ is the class probability of node *i* belonging to class *c*, *C* is the number of classes, ${\hat{y}}_{i}=\underset{c}{argmax}p_{ic}$ denotes a temporary label. The smaller entropy indicates a more reliable prediction. We focus on unlabeled nodes preliminarily assigned to the positive class, rank them by entropy ${ℋ}_{i}$ in ascending order, and retain a fixed number of lowest-entropy nodes to form a size-controlled candidate set.

However, relying solely on probabilities-based selection may lead to the inclusion of outlier candidates. Therefore, we impose the spectral clustering constraint based on the RBF kernel derived from the labeled positive set *S*_+_. Using standardized feature vectors ϕ(q) and ϕ(u), the RBF kernel with σ, and $\{C_{1},C_{2}\}\subseteq S_{+}$ denoting the two clusters, we construct $\kappa (q,u)=\exp\;\left(-\frac{\|\phi (q)-\phi (u){\|}_{2}^{2}}{2\sigma^{2}}\right)$ to perform spectral clustering, and compute the cluster centroids:


$$\begin{array}{c} \mu_{k}=\frac{1}{\left|C_{k}\right|}\sum_{i\in C_{k}}\phi (x_{i}),\;k=1,2. \end{array}$$ (8)

For each candidate unlabeled node *i*, we define its minimum distance to the positive cluster centroids as $d_{i}=\min_{k\in \{1,2\}}\|\phi (x_{i})-\mu_{k}{\|}_{2}$. By integrating entropy-based confidence filtering with the spectral clustering constraint based on the RBF kernel, the final selection rule for newly added positive samples is defined as:


$$\begin{array}{c} 𝒫=\{\;i\in 𝒰\;|\;{\hat{y}}_{i}=c^{+},\;p_{i,c^{+}}\geq \theta_{p},d_{i}\leq \mathrm{mean}\{d_{i}{\}}_{i\in S_{+}}+\mathrm{std}\{d_{i}{\}}_{i\in S_{+}}\}, \end{array}$$ (9)

where 𝒫 is the final pseudo-label set, 𝒰 is the set of unlabeled nodes, and *c*^+^ is the positive class. $p_{i,c^{+}}$ is the class probability that node *i* belongs to class *c*^+^, and $\theta_{p}\in (0,1)$ is the confidence threshold. Here, for a candidate unlabeled node, *d*_*i*_ represents its minimum distance to the positive cluster centroids, while $\mathrm{mean}\{d_{i}{\}}_{i\in S_{+}}$ and $\mathrm{std}\{d_{i}{\}}_{i\in S_{+}}$ denote the mean and standard deviation of the distances from the positive samples to the positive centroids.

#### Negative-sample inference.

Given the absence of an authoritative database of non-cancer driver genes, negatives are inferred from positives using the ICL model. Before negative inference, multiplex graphs contrastive pretraining is performed to obtain a consensus embedding matrix for node representations.

To maximize mutual information between local and global representations and learn more discriminative features, we compute a contrastive loss on each graph while aligning global representations via a cross-graph consensus regularization. We feed all graphs into the MDGCN to obtain the layer-wise node embeddings for the *m*-th graph as:


$$\begin{array}{c} H^{\left(m\right)}=\mathrm{Conv}(X,A^{\left(m\right)}). \end{array}$$ (10)

To aggregate node information at the graph level, we perform mean pooling over the positive nodes to obtain a graph-level vector *g*^(*m*)^ for each graph. Then, a shared bilinear discriminator is applied on each graph to construct positive and negative pairs for contrastive learning, and the resulting contrastive loss is given by:


$$\begin{array}{c} {ℒ}_{\text{contrast}}=-\frac{1}{n}\sum_{v}\log\sigma \;({\tilde{h}}_{v,+}^{\left(m\right)}M^{\left(m\right)}g^{\left(m\right)})-\frac{1}{n}\sum_{v}\log\sigma \;(1-{\tilde{h}}_{v,-}^{\left(m\right)}M^{\left(m\right)}g^{\left(m\right)}), \end{array}$$ (11)

where *M*^(*m*)^ is a learnable matrix, ${\tilde{h}}_{v,+}^{\left(m\right)}$ and ${\tilde{h}}_{v,-}^{\left(m\right)}$ denote the embeddings of node *v* in the *m*-th graph under the positive (i.e., original) and negative (i.e., corrupted) views, respectively. To establish consistent node representations across multiple graphs, we average the node embeddings from all graphs and introduce a learnable consensus embedding matrix $Z\in {\mathbb{R}}^{n\times f}$. The consensus loss is as follows:


$$\begin{array}{c} {ℒ}_{\text{consensus}}={\left\|Z-\frac{1}{M}\sum_{m=1}^{M}H_{+}^{\left(m\right)}\right\|}_{2}^{2}-{\left\|Z-\frac{1}{M}\sum_{m=1}^{M}H_{-}^{\left(m\right)}\right\|}_{2}^{2}, \end{array}$$ (12)

where $H_{+}^{\left(m\right)}$ and $H_{-}^{\left(m\right)}$ denote the node embeddings of the *m*-th graph under the positive and negative views. By combining all contrastive losses with the consensus loss, and using β to balance the two losses, the pretraining objective is:


$$\begin{array}{c} J=\sum_{m=1}^{M}{ℒ}_{\text{contrast}}+\beta {ℒ}_{\text{consensus}}. \end{array}$$ (13)

In addition, we introduce an ICL to infer high-confidence negatives. ICL is trained on positive samples only to model the internal consistency between each local window and its complementary segment. Samples with larger inconsistency are more likely to be negatives. For each sample $x_{i}\in {\mathbb{R}}^{f}$, we construct sliding window pairs $\Phi (x_{i})=\{(a_{i}^{j},b_{i}^{j})\overset{m}{\underset{j=1}{\}}}$ with *m* = *f* − *k* + 1, where $a_{i}^{j}\in {\mathbb{R}}^{k}$ is the *j*-th local window and $b_{i}^{j}\in {\mathbb{R}}^{f-k}$ is its complementary segment. After dual encoders *F* and *G* and normalization, we minimize the following objective under a contrastive learning framework with multiple candidates for each sample.


$$\begin{array}{c} \ell (F,G,\Phi (x_{i}),j)=-\ln\frac{\exp\;(F^{N}(b_{i}^{j})\cdot G^{N}(a_{i}^{j})/\tau )}{\sum_{j^{\prime }=1}^{m}\exp\;(F^{N}(b_{i}^{j})\cdot G^{N}(a_{i}^{j^{\prime }})/\tau )}, \end{array}$$ (14)

where *F*^*N*^ and *G*^*N*^ denote normalized embeddings, $j^{\prime }$ indexes candidate windows, and τ>0 is the temperature parameter. During inference, we construct $\Phi (x_{i})$ for a test sample *x* in the same manner and define the anomaly score as follows:


$$\begin{array}{c} y(x)=\sum_{j}\ell (F,G,\Phi (x_{i}),j). \end{array}$$ (15)

A higher *y*(*x*) indicates greater abnormality and therefore a higher probability that the sample is negative. After ranking the anomaly scores in descending order, we select the top-ranked samples in a quantity equal to the number of positives as the high-confidence negative set.

### Prediction of cancer driver genes

After *L* multiplex networks-based directed graph convolutional layers in the second MDGCN, the final representation *H*^(*L*)^ is fed into a linear classifier followed by softmax to obtain the predictive distribution:


$$\begin{array}{c} \hat{Y}=\mathrm{softmax}(H^{\left(L\right)}W_{cls}+b_{cls}), \end{array}$$ (16)

where *W*_*cls*_ and *b*_*cls*_ denote learnable classifier parameters.

#### Supervised loss.

Given the labeled node set $V_{L}\subseteq \{1,\ldots ,n\}$ and their labels $y_{i}\in \{0,1\}$, the cross-entropy loss is adopted:


$$\begin{array}{c} {ℒ}_{\text{sup}}=-\sum_{i\in V_{L}}\log{\hat{Y}}_{i,y_{i}}. \end{array}$$ (17)

#### Consistency regularization loss.

To mitigate learning abnormal weights that deviate from the distribution, we first average all weights associated with incoming and outgoing messages:


$$\begin{array}{c} {\bar{\gamma }}_{\to }=\frac{1}{n}\sum_{i=0}^{n-1}\Gamma_{(i,i),\to },\;{\bar{\gamma }}_{\leftarrow }=\frac{1}{n}\sum_{i=0}^{n-1}\Gamma_{(i,i),\leftarrow }, \end{array}$$ (18)

where $\Gamma_{(i,i),\to }$ is obtained by averaging the *i*-th diagonal entry of the diagonal matrix $\Gamma_{\to }^{\left(l,m\right)}$ over all graphs and layers. Then we minimize the distances between ${\bar{\gamma }}_{\to }$and $\Gamma_{(i,i),\to }$ and between ${\bar{\gamma }}_{\leftarrow }$ and $\Gamma_{(i,i),\leftarrow }$ using the following method.


$$\begin{array}{c} {ℒ}_{\text{reg}}=\frac{1}{n}\sum_{i=0}^{n-1}\overset{2}{\underset{2}{\left\|{\bar{\gamma }}_{\to }-\Gamma_{(i,i),\to }\right\|}}+\frac{1}{n}\sum_{i=0}^{n-1}\overset{2}{\underset{2}{\left\|{\bar{\gamma }}_{\leftarrow }-\Gamma_{(i,i),\leftarrow }\right\|}}. \end{array}$$ (19)

#### Objective function.

Finally, we combine the two losses above and use the hyperparameter λ to regulate the balance between them.


$$\begin{array}{c} ℒ={ℒ}_{\text{sup}}+\lambda {ℒ}_{\text{reg}}. \end{array}$$ (20)

### Implementation details of MNDGNN

Our model was implemented in Python 3.9.19 with PyTorch 2.1.2. We chose AdamW as the optimizer with a learning rate of 0.01. The MDGCN module comprised three layers (*L* = 3) with a hidden dimension of 256, a dropout rate of 0.5, and a weight decay of 0.0. We set α=0.5 to preserve information from the previous layer, and chose β=0.001 and λ=0.0003 to balance the contributions of the loss terms.

## Results

All the following experiments were conducted on a computer with an Nvidia RTX 4060 GPU. Each experiment was repeated with 10 different random seeds, with early stopping applied based on validation loss. The final reported results represent the average across all runs.

### Performance of MNDGNN in pan-cancer driver gene prediction

To evaluate the performance of MNDGNN, we compared our method with baseline models (GCN, GAT, SAGE, ChebNet, Dir-GNN, EMOGI, DGMP and MMGN) under ten runs of five-fold cross-validation (5CV) with area under the receiver operating characteristic curve (AUROC), area under the precision-recall curve (AUPRC) and F1 score metrics. For a fair comparison, all methods use the same multiplex biological networks and the same feature matrix. Methods limited to a single graph input operate on a merged graph constructed from the multiplex networks. Methods without negative sample inference are trained with the same positive and negative samples as ours. In addition, the hyperparameters of all baselines follow those specified in their original implementations.

As shown in Table 1, MNDGNN outperforms all competing methods across all evaluation metrics, indicating its superior accuracy in identifying cancer driver genes.

**Table 1:** Predictive performance of MNDGNN compared to other baseline models on multiplex networks.

Methods	AUROC	AUPRC	F1
GCN	0.8272	0.8494	0.7625
GAT	0.7729	0.7526	0.7545
SAGE	0.7959	0.8201	0.7393
ChebNet	0.8136	0.8292	0.7549
Dir-GNN	0.8202	0.8566	0.7614
EMOGI	0.8360	0.8567	0.7766
DGMP	0.8396	0.8585	0.7911
MMGN	0.8634	0.8845	0.7981
MNDGNN	0.8780	0.8962	0.8238

### Ablation experiments

To validate the contribution of different biological networks, we first conducted ablations of input networks on the pan-cancer dataset under 5CV. We removed one network at a time and trained the model using the remaining biological networks. To analyze the contributions of different components in MNDGNN, we subsequently performed directionality and data augmentation ablations. “Without directionality” means all graphs are treated as undirected. “Without positive-sample augmentation” means the positive-sample augmentation module is disabled. “Without negative-sample inference” means that we use the EMOGI negative set and randomly sample an equal number of negative genes to match the positives during training.

As shown in Table 2, our full model achieves the best performance in AUROC, AUPRC and F1 score compared with its variants. Removing any single biological network consistently degrades performance, suggesting that each network provides useful information for cancer driver gene identification. In particular, excluding the PPI network or DawnNet leads to the largest drops, indicating these two networks capture especially important informative relationships. When edge directionality is removed, AUROC decreases by approximately 1.7%, AUPRC by approximately 1.8%, and F1 score by nearly 3%, underscoring the importance of directionality in networks such as gene regulation. Disabling either positive-sample augmentation or negative-sample inference also reduces performance, further supporting the utility of the proposed data augmentation strategy. Overall, these ablations show that both diverse biological network inputs and the components of MNDGNN contribute to performance, validating the effectiveness of our framework for cancer driver gene identification.

**Table 2:** The ablation experimental results of MNDGNN on multiplex networks in 5CV test.

Methods	AUROC	AUPRC	F1
Without PPI network	0.8447	0.8487	0.7947
Without protein complexes network	0.8654	0.8839	0.8137
Without KEGG pathway network	0.8661	0.8837	0.7969
Without RegNetwork	0.8703	0.8872	0.8042
Without DawnNet	0.8566	0.8756	0.8001
Without kinase-substrate network	0.8654	0.8781	0.8113
Without directionality	0.8609	0.8778	0.7944
Without positive-sample augmentation	0.8767	0.8890	0.8062
Without negative-sample inference	0.8544	0.8625	0.8018
MNDGNN	0.8780	0.8962	0.8238

### Performance on independent test sets

To assess whether the model is biased toward a specific data source, we evaluated its generalization on two independent test sets derived from OncoKB [30] and ONGene [31]. For each independent set, we first removed genes that overlap with the training positives. Under this setup, genes in the independent set were treated as true positives, and all remaining genes outside this set were treated as negatives. We then computed the AUPRC for each method and did not perform any hyperparameter tuning on the independent set.

**Fig 2:**
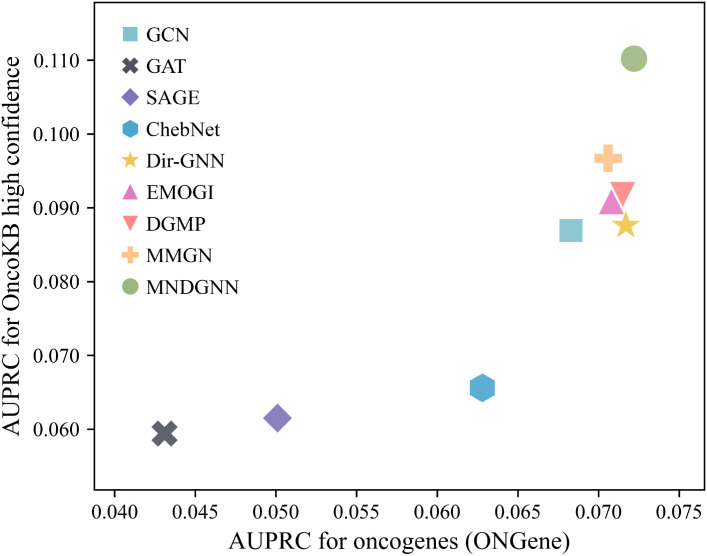
Performance comparison of different methods on OncoKB and ONGene.

All methods exhibit relatively low AUPRC on the independent test sets due to the limited number of true positives. Nevertheless, MNDGNN achieves the highest AUPRC on both OncoKB and ONGene (Fig 2), validating its robustness and ability to generalize beyond any single curated dataset.

### Performance under class imbalance

**Fig 3:**
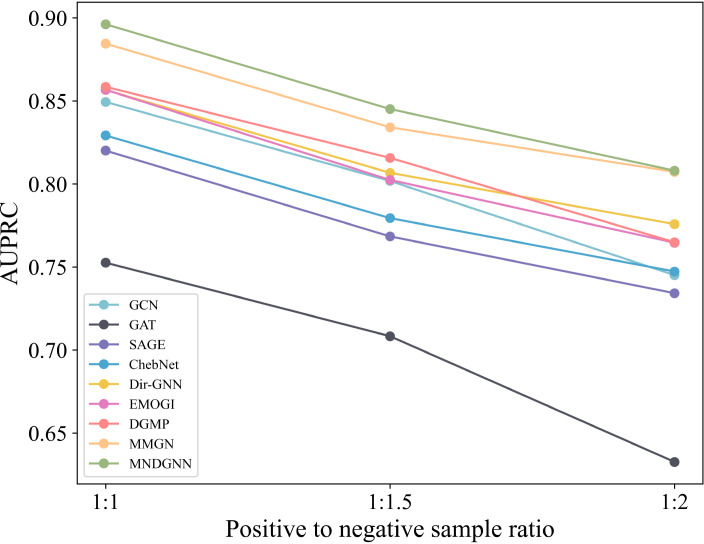
Performance comparison of different methods under different positive-to-negative sample ratios.

During the data augmentation stage, we used the same number of negative and positive samples to mitigate class imbalance. To further evaluate the effect of the positive-to-negative sample ratio on model performance, we set three ratios, namely 1:1, 1:1.5, and 1:2, while keeping all the other experimental data and parameters unchanged. All comparative methods were evaluated under these settings. We selected AUPRC as the evaluation metric and plotted a line chart (Fig 3) to directly compare the performance changes of different models under different sample ratios.

The results show a clear downward trend in AUPRC for all models as the proportion of negative samples increases. This indicates that class imbalance weakens the model’s identification capability. Specifically, each model achieves its best performance at the 1:1 ratio. This suggests that a relatively balanced sample distribution is more conducive to learning discriminative features of the positive class. As the number of negative samples increases, the models become more likely to be dominated by the majority class, thereby reducing their ability to identify positive samples. In addition, although performance declines under class-imbalanced settings, our model still achieves the best results, demonstrating superior stability and robustness.

### Enrichment analysis

**Fig 4:**
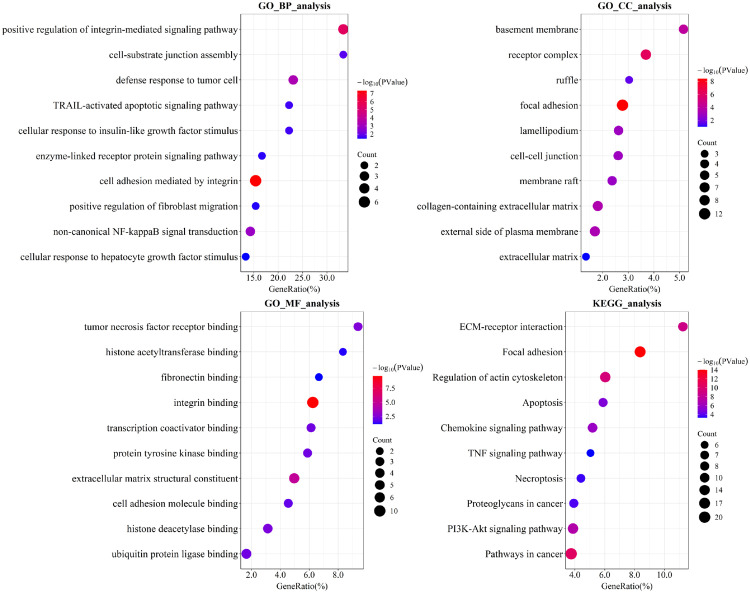
Enrichment analysis of the top 50 newly predicted CDGs included GO categories (BP, CC, MF) and KEGG pathway enrichment analysis.

We applied the trained model to the remaining unlabeled genes after removing training genes, ranked the candidates by their predicted probability of being CDGs, and selected the top 50 newly predicted CDGs for GO and KEGG pathway enrichment analysis. Fig 4 summarizes the enrichment results. In each bubble plot, the x-axis denotes the proportion of genes annotated to a pathway, bubble color indicates statistical significance, and bubble size reflects the number of hit genes.

Regarding biological processes (BP), the genes exhibit significant enrichment in the positive regulation of integrin-mediated signaling pathway, cell-substrate junction assembly, cell adhesion mediated by integrin, TRAIL-activated apoptotic signaling pathway, and non-canonical NF-kappaB signaling transduction. In the context of cellular components (CC), the genes are predominantly localized to migratory and adhesive structures such as focal adhesion, lamellipodium, and ruffle; receptor platforms including membrane raft and receptor complex; as well as matrix and barrier components like the basement membrane, collagen-containing extracellular matrix, extracellular matrix (ECM) and external side of the plasma membrane. For molecular functions (MF), the functional activities are concentrated on ECM-receptor interfaces and transcriptional or epigenetic regulation, encompassing integrin binding, fibronectin binding, cell adhesion molecule binding, protein tyrosine kinase binding, tumor necrosis factor receptor binding, ubiquitin protein ligase binding, histone acetyltransferase binding, histone deacetylase binding, and transcription coactivator binding. In terms of KEGG pathway, the genes are primarily enriched in ECM-receptor interaction, focal adhesion, regulation of actin cytoskeleton, apoptosis, necroptosis, proteoglycans in cancer, TNF signaling pathway, PI3K-Akt signaling pathway and pathways in cancer.

Collectively, these results indicate the identified driver genes are closely associated with known cancer pathways and may play key roles in tumor initiation and progression.

### Interaction analysis between newly predicted CDGs and known CDGs

We conducted an in-depth analysis of the newly predicted CDGs. The model score, interpreted as the predicted probability that a gene is a cancer driver gene, shows a significant association with the number of interactions with known CDGs, as quantified by Spearman’s rank correlation. Since our dataset contains both undirected and directed graphs, we consider three interaction types for each gene: **in** (in-degree interactions), **out** (out-degree interactions), and **total**. Specifically, **total** denotes the count of unique neighbors among known CDGs for a given gene, regardless of edge direction. It is computed by collecting all known CDGs linked to the gene in the graph and counting the unique entries after deduplication. For undirected graphs, these three counts are identical. For directed graphs, **total** is defined as the size of the union of in-neighbors and out-neighbors. If the same known CDG appears in both directions, it is counted only once.

**Fig 5:**
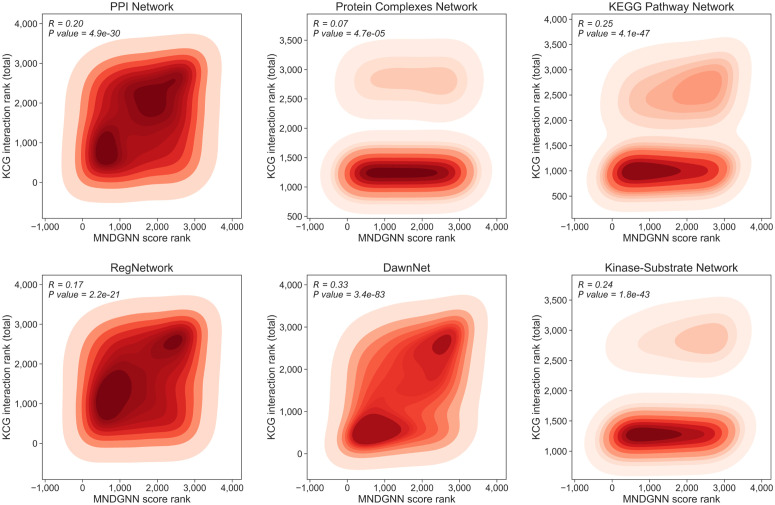
Spearman correlation and bivariate kernel density between the MNDGNN prediction score rank and the rank of the total number of interactions with known CDGs across different networks.

For total interaction counts, we plot rank correlations between each gene’s score (predicted probability) and its number of interactions with known CDGs across the six networks. Genes with higher scores tend to interact with more known CDGs. As shown in Fig 5, the strongest correlation appears in the DawnNet, followed by the KEGG pathway network and kinase-substrate network. The PPI network is dense and undirected, exhibiting a significant yet broadly dispersed correlation. The RegNetwork is weaker but still significant. The protein complexes network is the weakest, likely limited by noisier data. Overall, the density contour lines shift toward the upper right as the score increases, indicating high-scoring genes interact with more known CDGs.

**Fig 6:**
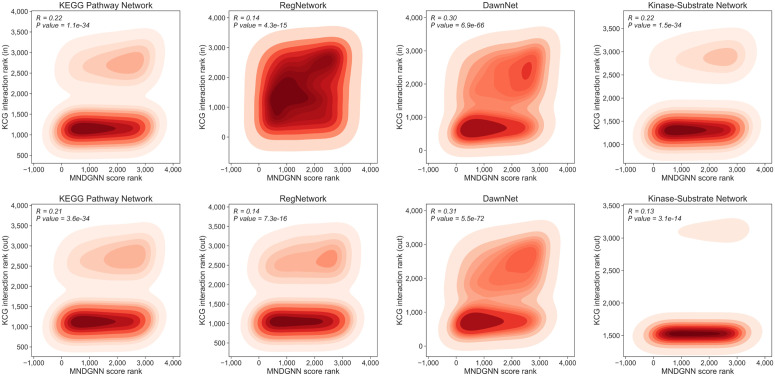
Spearman correlation and bivariate kernel density between the MNDGNN prediction score rank and the ranks of incoming and outgoing interaction counts with known CDGs in directed networks.

For directed graphs, we compute not only total interactions, but also incoming and outgoing interaction counts. In Fig 6, the DawnNet shows strong correlations in both directions, indicating enrichment for both upstream regulation by known CDGs and downstream effects. The KEGG pathway network yields similar values in the two directions. Pathway edges are directed reactions, and both directions capture path proximity to known CDGs, so the correlations are comparable. The kinase-substrate network is asymmetric, with the incoming directions stronger than the outgoing, suggesting that kinases pointing to substrates drive the trend more. The RegNetwork is weaker in both directions yet remains significant. Directionality reveals concordance or asymmetry between incoming and outgoing interactions, which strengthens the biological interpretability of the model’s predictions.

### Drug sensitivity analysis

**Fig 7:**
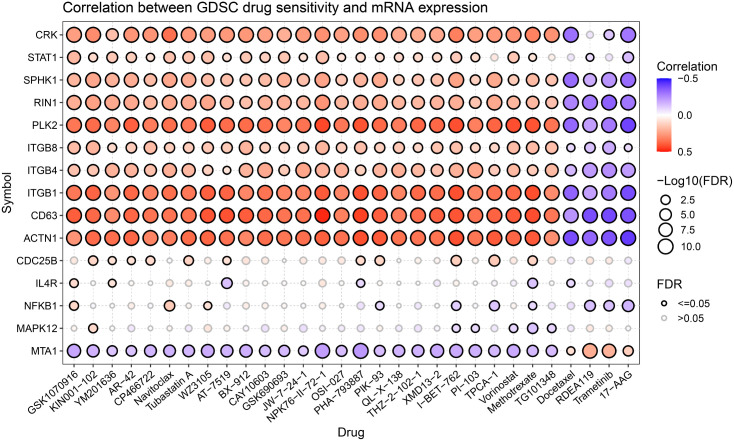
Correlation between GDSC drug sensitivity and mRNA expression for the top 15 newly predicted CDGs.

We selected the top 15 newly predicted cancer driver genes and conducted drug sensitivity analysis on the GDSC via Gene Set Cancer Analysis [32]. As shown in Fig 7, most predicted genes exhibit significant associations with sensitivity to multiple classes of clinically relevant targeted therapies. This suggests that most predicted genes indeed occupy key signaling nodes in mediating drug response. The GDSC drug sensitivity analysis supports, from a pharmacological perspective, the biological plausibility and accuracy of the cancer driver genes predicted by our model, and provides a credible basis for subsequent target validation and therapeutic strategy design.

For example, GSK1070916 selectively inhibits Aurora B/C, thereby blocking mitosis and inducing apoptosis, and exhibits broad antitumor activity [33]. YM201636 promotes epidermal growth factor receptor expression by inducing autophagy, thereby suppressing tumor growth [34]. AT-7519 induces cell death through multiple pathways and inhibits glioblastoma progression [35]. CAY10603 ameliorates diabetic nephropathy by suppressing NLRP3 inflammasome activation in renal tubular cells and macrophages [36]. OSI-027 overcomes rapamycin insensitivity via dual inhibition of mTORC1/2 and induces tumor cell death through activation of the PI3K-AKT signaling pathway [37]. PIK-93 promotes PD-L1 ubiquitination, and in combination with anti-PD-L1 antibodies enhances T-cell activation to inhibit tumor growth [38]. QL-X-138 suppresses B-cell malignancies through dual inhibition of BTK/MNK, arresting lymphoma and leukemia cells in G0-G1 phase [39]. Finally, TPCA-1 concurrently inhibits NF-κB and STAT3 signaling in lung cancer and, in combination with tyrosine kinase inhibitors, synergistically treats non-small cell lung cancer [40].

## Discussion and conclusion

In this study, we propose a model named MNDGNN for identifying cancer driver genes. Specifically, we design the MDGCN module as a stack of multiplex networks-based directed graph convolutional layers that integrate neighbor diversity and degree diversity. In each layer, edge directionality is modeled through learnable node-level weights, enabling the network to capture subtle differences among gene nodes. Furthermore, each layer fuses information from multiplex graphs, allowing the module to fully exploit the rich information contained in diverse biological networks. In addition, we augment positives using low information entropy and RBF spectral clustering, and we introduce the ICL to infer high-confidence negatives from positives, which mitigates label scarcity and overfitting. Finally, we train the model on the augmented data and use a linear classifier to predict candidate cancer driver genes.

The results demonstrated superior performance and stable effectiveness of the proposed model, as evidenced by comparative, ablation, and independent test set experiments on multiplex biological networks. Model predictions were cross-validated at multiple levels, highlighting its robustness across hierarchies. The top 50 newly predicted cancer driver genes were subjected to GO and KEGG enrichment analyses, revealing mechanistic associations between these predicted genes and cancer pathways. We further validated biological interpretability by examining the relationship between model scores and interactions with known CDGs, showing that high-scoring genes exhibited stronger topological proximity and functional relatedness in the relevant networks. In addition, drug sensitivity analyses based on the top 15 newly predicted cancer driver genes revealed significant associations between the majority of these genes and multiple compounds, supporting their biological plausibility and predictive accuracy and informing subsequent therapeutic strategy design.

Despite its strong performance in pan-cancer driver gene prediction, our model still has several limitations. Incomplete networks and noisy edges may affect performance. Moreover, different biological networks are likely to contribute unequally, yet in this work we adopt simple averaging to combine them, implicitly treating all networks as equally informative. Future work will focus on improving robustness to network noise and learning adaptive cross-network integration, for example by incorporating attention-based fusion or related weighting mechanisms to assign specific importance to each network and thereby further enhance model performance.

## Supporting information

S1 File The list of the top 50 predicted cancer driver genes. (DOCX)
