# Supplementary material for: Multiplex networks-based directed graph neural network for cancer driver gene identification
Source: PLoS Comput Biol. 2026 May 14;22(5):e1014275. doi: 10.1371/journal.pcbi.1014275 (PMC13175473; doi:10.1371/journal.pcbi.1014275)
Supplement: S1 File — (DOCX) [file pcbi.1014275.s001.docx]

**Supplementary file of “Multiplex networks-based directed graph neural network for cancer driver gene identification”**

**1. Top 50 predicted cancer driver genes**

Tabel 1 Top 50 predicted cancer driver genes

| 1. NFKB1 | 2. STAT1 | 3. PLK2 | 4. ITGB1 | 5. RIN1 |
| --- | --- | --- | --- | --- |
| 6. CDC25B | 7. IL4R | 8. ITGB8 | 9. MAPK12 | 10. CRK |
| 11. SPHK1 | 12. ACTN1 | 13. MTA1 | 14. CD63 | 15. PXN |
| 16. ITGB4 | 17. CD4 | 18. LAMB3 | 19. CTSB | 20. TRAF2 |
| 21. ANAPC1 | 22. STAT2 | 23. LIMK1 | 24. STAT4 | 25. ITGB3 |
| 26. TUBA4A | 27. TNFRSF10B | 28. ITGB5 | 29. LTB | 30. RAC2 |
| 31. TGM2 | 32. FN1 | 33. GPI | 34. LAMB2 | 35. ITGB2 |
| 36. BCAR1 | 37. SPI1 | 38. DYNC1H1 | 39. GRB2 | 40. LAMC1 |
| 41. DOCK1 | 42. LAMA1 | 43. PI4KB | 44. GNAI2 | 45.RELA |
| 46. STAT5A | 47. MCM5 | 48. SP1 | 49. TNFRSF1A | 50. MAP3K5 |
